# CD163 as a marker of M2 macrophage, contribute to predict aggressiveness and prognosis of Kazakh esophageal squamous cell carcinoma

**DOI:** 10.18632/oncotarget.15630

**Published:** 2017-02-22

**Authors:** Jian Ming Hu, Kai Liu, Ji Hong Liu, Xian Li Jiang, Xue Li Wang, Yun Zhao Chen, Shu Gang Li, Hong Zou, Li Juan Pang, Chun Xia Liu, Xiao Bin Cui, Lan Yang, Jin Zhao, Xi Hua Shen, Jin Fang Jiang, Wei Hua Liang, Xiang Lin Yuan, Feng Li

**Affiliations:** ^1^ Department of Pathology and Key Laboratory of Xinjiang Endemic and Ethnic Diseases (Ministry of Education), Shihezi University School of Medicine, Shihezi, 832003, China; ^2^ Department of Pathology, Beijing Chaoyang Hospital, Capital Medical University, Beijing, 100020, China; ^3^ Department of Oncology, Tongji Hospital, Tongji Medical College, Huazhong University of Science and Technology, Wuhan, 430030, China; ^4^ Department of Preventive Medicine, Shihezi University School of Medicine, Shihezi, 832003, China

**Keywords:** Kazakh, esophageal squamous cell carcinoma, macrophage, CD163, MMP9

## Abstract

M2 macrophages was domesticated by tumor microenvironment to produce some angiogenic molecules and protease, facilitating angiogenesis and matrix breakdown, promoting tumor invasive and metastasis. However, The function of M2 macrophages to progression of esophageal carcinoma, especially Kazakh esophageal carcinoma is still dimness. This study aims to investigate M2 macrophages correlated with matrix metalloproteinase-9 (MMP9) and microvessel density, and the role in the progression of Kazakh esophageal squamous cell carcinoma. CD163 and CD34 as the marker of M2 macrophages and endothelial cells, were used to identify the M2 macrophages density and microvessel density, respectively. Immunohistochemistry staining was evaluated the expression of MMP9. The number of infiltrated CD163-positive M2 macrophages in tumor islets and stroma was significantly higher than in cancer adjacent normal tissues. The increased of M2 macrophages and microvessel density were significantly correlated with more malignant phenotypes including lymph node metastasis and clinical stage progression. Meanwhile, the expression of MMP9 showed much higher level in esophageal squamous cell carcinoma than that in cancer adjacent normal tissues, and high expression of MMP9 in Kazakh esophageal squamous cell carcinoma was significantly associated with age, depth of tumor invasion, lymph node metastasis, and tumor clinical stage. The quantity of M2 macrophages in tumor stroma was positively associated with microvessel density and the expression of MMP9, and as an independent poorly prognostic factor for overall survival time of Kazakh esophageal squamous cell carcinoma. These findings suggest the increased number of M2 macrophages correlated with high expression of MMP9 and high microvessel density may contribute to the tumor aggressiveness and angiogenesis, promoting the progression of Kazakh esophageal squamous cell carcinoma.

## INTRODUCTION

Esophageal carcinoma is one of most common malignant tumors in the world. According to its etiological and pathological characteristics, it is divided into two main forms: esophageal squamous cell carcinoma (ESCC) and esophageal adenocarcinoma. More than 90% of esophageal cancers are classified as ESCCs in China [1]. The Kazakh national minority (ethnic) living in Xinjiang (northwest of China) is a demographic with one of the highest rates of esophageal carcinoma incidence and mortality in China [2]. The 5-year survival rate of esophageal carcinoma is only 10%. Primary reasons for poor prognosis are associated with early stage cancer cell invasion and high metastasis [3].

The tumor microenvironment is important for cancer development and metastasis [4, 5]. It contains a range of inflammatory and immune cells. Macrophages are essential immune cells that play a critical role in carcinogenesis and tumor progression [6]. Mirroring T helper type 1, T helper type 2 (TH1, TH2) polarization, two distinct states of polarized activation for macrophages have been recognized: the classically activated (M1) macrophage phenotype and the alternatively activated (M2) macrophage phenotype [7, 8]. Bacterial moieties such as LPS and the interferon-γ (IFN-γ) polarize macrophages toward the M1 phenotype, showed more proinflammatory and antitumor activity, high expression of tumor necrosis factor alpha [TNF-α, interleukin [IL]-6, IL-12) and major histocompatibility complex (MHC-II) molecules. In contrast, IL-4, IL-10, and IL-13 polarize macrophages toward the M2 phenotype, showed more phagocytic activity and carcinogenesis function, high expression of IL-10 and arginase, but low expression of MHC class II and IL-12 [9]. Most M2 macrophages are considered to be tumor associated macrophages (TAMs) and function to promote tumor angiogenesis and metastasis [10]. One of the mechanism is M2 macrophages which could produce growth factors and proteases that degrade the tissue extracellular matrix (ECM), and facilitate the tumor cells escape from their constraining basement membranes [11]. In this procession, matrix metalloproteinases(MMPs), particularly MMP9, which is delivered into the tumor microenvironment by M2 macrophages, favoring tumor stromal angiogenesis [12], and other study also showed M2 macrophages produces many proangiogenic factors and expresses high levels of MMP9, which promote cancer cell invasion, metastasis and angiogenesis [13]. However, the precise role of M2 macrophage in Kazakh esophageal carcinoma has yet to be elucidated. So we used CD163 and CD34 as marker of M2 macrophages and endothelial cells to assay the M2 macrophages density and microvessel density (MVD) separately, then we used immunohistochemistry(IHC) to evaluate the MMP9 expression. Ultimately, we aim to investigate whether the increased number of M2 macrophages was correlated with high expression of MMP9 and high MVD, which contributes to the tumor aggressiveness and angiogenesis, and facilitates the occurrence and progression of Kazakh ESCC.

## RESULTS

### Distributions of CD163-positive M2 macrophages in Kazakh ESCCs and the correlation of M2 macrophages density with Kazakh ESCC clinicopathological parameters

**Figure 1:**
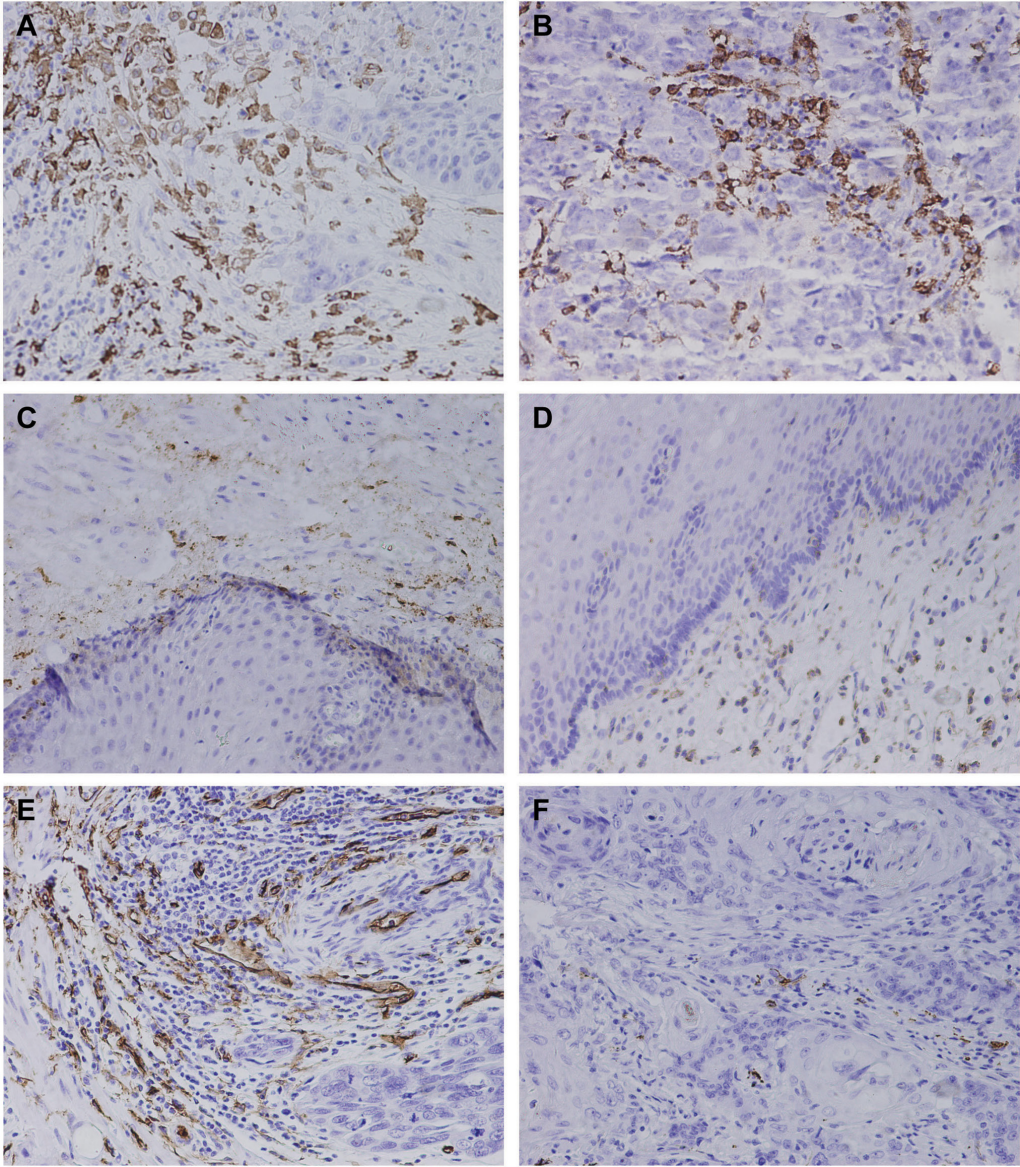
The distribution of CD163-positive M2 macrophae and microvessel in Kazakh esophageal squamous cell carcinoma (ESCC) and Cancer adjacent normal (CAN) tissues (**A**–**D**) Immunohistochemical staining of CD163, which was used as a marker of M2 macrophages and to evaluate the density of M2 macrophages in ESCC and CAN tissues. (A) and (B) showed the distribution of M2 macrophages in ESCC tumor stromal and islet, CD163 revealed diffuse staining of membranes and cytoplasm of M2 macrophages, and showed the high density of M2 macrophages located in ESCC tissues (especially in tumor stroma). (C) and (D) showed the distribution of M2 macrophages in CAN stroma and epithelia. A small number of CD163-positive M2 macrophages appear in CAN tissues. (**E** and **F**) Immunohistochemical staining of CD34, which was used to mark endothelial cells and to evaluate the microvessel density (MVD) in ESCC and CAN tissues. (E) showed high MVD in Kazakh ESCC tissues. (F) showed low MVD in Kazakh ESCC tissues.

We used CD163 as a marker to assess M2 macrophages distribution. Immunohistochemistry staining for CD163 revealed diffuse staining of M2 macrophages membranes and cytoplasm (Figure 1). We found that M2 macrophages were primarily located in the tumor stroma, but a small number of M2 macrophages reside in tumor islets. The density of M2 macrophages in Kazakh ESCC tumor islets (approximately 15/HPF, 0–45) and stroma (approximately 58/HPF, 9–139) were significantly higher compared to cancer adjacent normal (CAN) epithelia (approximately 2/HPF, 0-10) and stroma (approximately 19/HPF, 3–54) (all *P* < 0.001, Table 1). To explore the role of M2 macrophages in progression of Kazakh ESCCs, we divided the cases into high and low density of M2 macrophage groups according to the median value of CD163-positive macrophages (15 for tumor islet and 58 for tumor stroma), and evaluated possible correlations between M2 macrophages density and Kazakh clinicopathological parameters, including age, gender, tumor location, histological grade, invasion depth, nodal status, and clinical stages, found the increased number of M2 macrophages in tumor stroma were significantly correlated with more malignant phenotypes of Kazakh ESCCs, including lymph node metastasis and clinical stage progression (all *P* = 0.001). Similar, high density of M2 macrophages in tumor islet also were correlated with lymph node metastasis of ESCCs (*P* < 0.05). No significant correlations were found between M2 macrophages distribution and other parameters of Kazakh ESCCs (*P* > 0.05, Table 2).

**Table 1:** The distribution of CD163-positive macrophages in Kazakh esophageal squamous cell carcinoma (ESCC) and Cancer adjacent normal (CAN) tissues

Groups	Cases (*N*)	Isletscount range)	*Z/P*	Stroma(count range)	*Z/P*
**ESCCs**	100	15 (0–45)	11.559/0.000*	58 (9–139)	10.081/0.000*
**CANs**	100	2 (0–10)		19 (3–54)	

^*^P < 0.05.

**Table 2:** Tumor infiltrating CD163-positive macrophages in Kazakh esophageal squamous cell carcinoma (ESCC) islets and stroma, and their correlation with clinicopathological parameters

Variable	Cases(*N*)	CD163+ macrophage counts in tumor islets		*P*	CD163+ macrophage counts in tumor stroma		*P*
		Low (< 15)	High (≥ 15)		Low (< 58)	High (≥ 58)	
**Age (y)**							
≤Median(58 y)	53	34 (64.2%)	19 (35.8%)	1.000	31 (58.5%)	22 (41.5%)	0.164
> Median	47	31 (66.0%)	16 (34.0%)		20 (42.6%)	27 (57.4%)	
**Gender**							
Male	68	47 (69.1%)	21 (30.9%)	0.301	35 (51.5%)	33 (48.5%)	1.000
Female	32	18 (56.3%)	14 (43.8%)		16 (50.0%)	16 (50.0%)	
**Tumor location**							
Upper	2	2 (100.0%)	0 (0.00 %)	0.272	1 (50.0%)	1 (50.0%)	0.991
Middle	70	47 (67.1%)	23 (32.9%)		36 (51.4%)	34 (48.6%)	
Lower	28	16 (57.1%)	12 (42.9%)		14 (50.0%)	14 (50.0%)	
**Histologic grade**							
Well	29	21 (72.4%)	8 (27.6%)	0.545	15 (51.7%)	14 (48.3%)	0.838
Moderate	47	30 (63.8%)	17 (36.2%)		25 (53.2%)	22 (46.8%)	
poor	24	14 (58.3%)	10 (41.7%)		11 (45.8%)	13 (54.2%)	
**Depth of invasion**							
TI–T2	35	19 (54.3%)	16 (45.7%)	0.153	20 (57.1%)	15 (42.9%)	0.489
T3–T4	65	46 (70.8%)	19 (29.2%)		31 (47.7%)	34 (52.3%)	
Nodal **status**							
pN–	51	39 (76.5%)	12 (23.5%)	0.025*	35 (68.6%)	16 (31.4%)	0.001*
pN+	49	26 (53.1%)	23 (46.9%)		16 (32.7%)	33 (67.3%)	
**Clinical stage**							
I–II	62	42 (67.7%)	20 (32.3%)	0.604	40 (64.5%)	22 (35.5%)	0.001*
III–IV	38	23 (60.5%)	15 (39.5%)		11 (28.9%)	27 (71.1%)	

Abbreviations: pN−, no lymph node metastasis; pN+: node metastasis. *P < 0.05.

### Distributions of microvessel in Kazakh ESCCs and the correlation of microvessel density (MVD) with Kazakh ESCC clinicopathological parameters

CD34 staining was used to assess MVD in tumor tissues. Immunohistochemistry staining for CD34 revealed diffuse staining of endothelial cells membranes and cytoplasm (Figure 1). The mean value of MVD in Kazakh ESCCs was 17 per high-power field (range, 3–31), which was significantly higher compared to CAN (mean, 9/HPF; range, 2–19) (Supplementary Figure 1). Similar to classify of M2 macrophages density, we divided the cases into high and low density of microvessel groups according to the median value of MVD, and we found high MVD in Kazakh ESCCs was significantly associated with more malignant phenotypes including lymph node metastasis and clinical stage progression (all *P* < 0.05) (Table 3).

**Table 3:** Correlation between microvessel density (MVD) and clinicopathological parameters in Kazakh esophageal squamous cell carcinoma (ESCC) tumor stroma

Variable	Cases(*N*)	MVD		*P*
		Low (< 17)	High (≥ 17)	
**Age (y)**				
≤ Median (58y)	53	28 (52.8%)	25 (47.2%)	0.540
> Median	47	21 (44.7%)	26 (55.3%)	
**Gender**				
Male	68	31 (45.6%)	37 (54.4%)	0.435
Female	32	18 (56.2%)	14 (43.8%)	
**Tumor location**				
Upper	2	0 (0.0%)	2 (100.0 %)	0.255
Middle	70	35 (50.0%)	35 (50.0%)	
Lower	28	14 (50.0%)	14 (50.0%)	
**Histologic grade**				
Well	29	15 (51.7%)	14 (48.3%)	0.197
Moderate	47	26 (55.3%)	21 (44.7%)	
poor	24	8 (33.3%)	16 (66.7%)	
**Depth of invasion**				
TI–T2	35	19 (54.3%)	16 (45.7%)	0.571
T3–T4	65	30 (46.2%)	35 (53.8%)	
Nodal **status**				
pN−	51	31 (60.8%)	20 (39.4%)	0.027*
pN+	49	18 (36.7%)	31 (63.3%)	
**Clinical stage**				
I–II	62	36 (58.1%)	26 (41.9%)	0.035*
III–IV	38	13 (34.2%)	25 (65.8%)	

Abbreviations: pN^−^, no lymph node metastasis; pN^+^: node metastasis. *P < 0.05.

### MMP9 expression in Kazakh ESCCs and CANs, and its relationship with ESCC clinicopathological parameters

**Figure 2:**
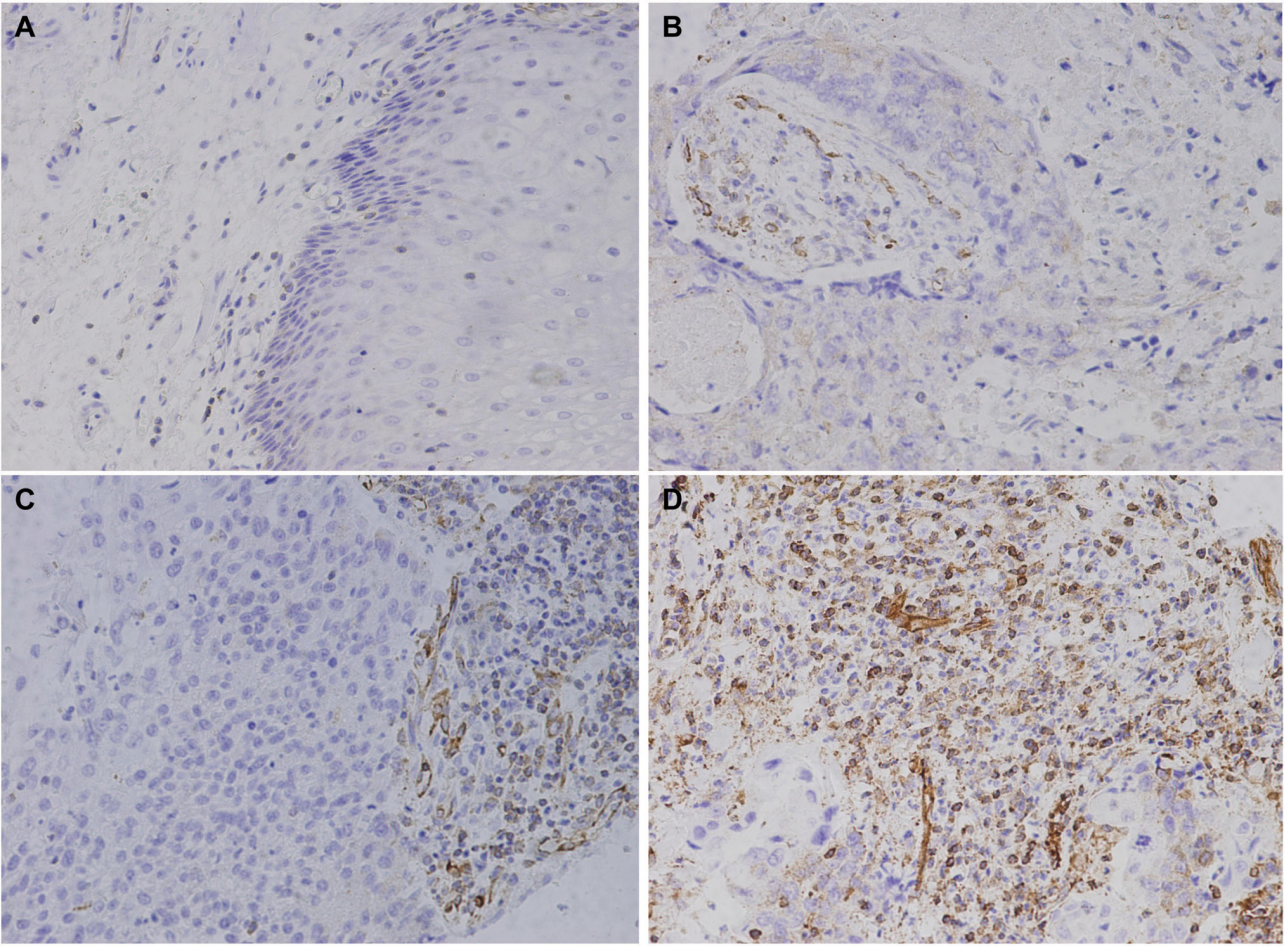
Immunohistochemical staining of MMP9 in Kazakh esophageal squamous cell carcinoma (ESCC) and Cancer adjacent normal (CAN) tissues MMP9 staining is primarily observed in tumor stroma (cell membranes and cytoplasm), some ESCC cells also show staining. (**A**) Negative MMP9 staining is shown in CAN tissues (scored as 0). (**B**) Weak MMP9 staining is shown in ESCC tissues (scored as 1). (**C**) and (**D**) show moderate and strong MMP9 staining in Kazakh ESCC tissues, respectively (scored as 2 and 3, respectively).

M2 macrophages are reported to be involved in tumor growth and metastasis, and are suspected to be important for tumor progression [14]. MMP-9 is delivered into the tumor microenvironment by M2 macrophages, and subsequently sustains those distinct angiogenic vessels capable, supporting tumor cell intravasation and metastatic dissemination [15]. So we decided to investigate whether MMP9 is the key molecule mediated by M2 macrophages to play an important role in the occurence and progression of Kazakh ESCC. As shown in Figure 2, MMP9 staining was mainly observed in tumor stromal cells (located in cell membranes and cytoplasm), including macrophages, ESCC tumor cells and endothelial cells (ECs). We compared three categories of MMP9 positive staining combinations (1+, 2+/3+, and 1+/2+/3+) to MMP9 negative (0) staining (Table 4). The MMP9 positive rate (1+/2+/3+) in ESCC was higher than that in CAN tissues (90.0% vs 56.0%; *P* < 0.001). Differences in MMP9 positive rates from ESCC to CAN tissues were more prominent in strong staining cases (2+/3+; 55.0% vs 16.0%; *P* < 0.001).

**Table 4:** The expression of MMP9 in Kazakh esophageal squamous cell carcinoma (ESCC) and cancer adjacent normal (CAN) tissues

Characteristics	N	Negative	Positive combination								
		0 (%)	1+(%)	X^2^	P	2+3+(%)	X^2^	P	1+2+3+	X^2^	P
ESCCs	100	10 (10.0%)	35 (35.0%)	9.393	0.002*	55 (55.0%)	40.369	0.000*	90 (90.0%)	27.625	0.000*
CANs	100	44 (44.0%)	41 (41.0%)			16 (16.0%)			56 (56.0%)		

^*^P < 0.05.

In order to investigate the role of MMP9 in the progression of Kazakh ESCCs, we divided cases into two categories according to MMP9 expression level: low expression (−/1+) and high expression (2+/3+). The expression level of MMP9 was significantly higher in over-58-year old patients compared to under-58-years patients (68.1% vs 43.4%; *P* < 0.05). Cases with high MMP9 expression showed strong invasion (T3–T4 vs T1–T2 = 63.1% vs 40.0%; *P* < 0.05) and metastasis (pN+ vs pN− =73.5% vs 37.7%; *P* = 0.001), and were clearly present in advanced ESCC stages (III–IV vs I–II = 78.9% vs 40.3%; *P* < 0.001) (Table 5).

**Table 5:** Correlation between expression of MMP9 and clinicopathological parameters in Kazakh esophageal squamous cell carcinoma (ESCC) tissues

Variable	Cases (*N*)	MMP9 low expression	MMP9 high expression	X^2^	*P*
		0/1+(%)	2+/3+(%)		
**Age (y)**					
≤ Median (58y)	53	30 (56.6%)	23 (43.4%)	5.178	0.023*
> Median	47	15 (31.9%)	32 (68.1%)		
**Gender**					
Male	68	30 (44.1%)	38 (55.9%)	0.002	0.966
Femle	32	15 (46.9%)	17 (53.1%)		
**Tumor location**					
Upper	2	2 (100.0%)	0 (0.0%)	2.597	0.273
Middle	70	30 (42.9%)	40 (57.1%)		
Lower	28	13 (46.4%)	15 (53.6%)		
**Histologic grade**					
Well	29	10 (34.5 %)	19 (65.5%)	2.699	0.259
Moderate	47	25 (53.2%)	22 (46.8%)		
poor	24	10 (41.7%)	14 (58.3%)		
**Depth of invasion**					
TI–T2	35	21 (60.0%)	14 (40.0%)	4.007	0.045*
T3–T4	65	24 (36.9%)	41 (63.1%)		
Nodal **status**					
pN−	51	32 (62.7%)	19 (37.7%)	11.819	0.001*
pN+	49	13 (26.5%)	36 (73.5%)		
**Clinical stage**					
I–II	62	37 (59.7%)	25 (40.3%)	12.684	0.000*
III–IV	38	8 (21.8%)	30 (78.9%)		

Abbreviations: pN^−^, no lymph node metastasis; pN^+^: node metastasis. *P < 0.05.

### Correlation between the density of M2 macrophages, microvessel density (MVD) and MMP9 expression in Kazakh ESCCs

**Figure 3:**
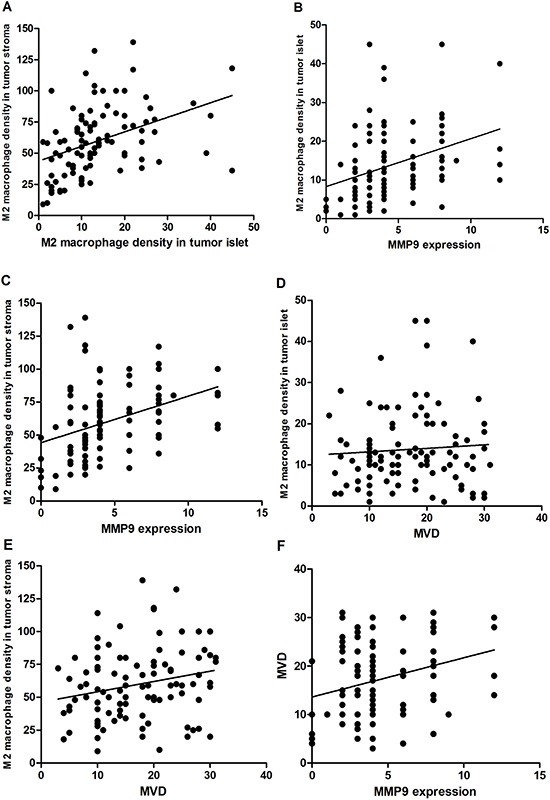
Cross correlation analyses reveal strong relationships among the density of M2 macrophage in tumor islet, tumor stroma and expression of MMP9, microvessel density (MVD) in Kazakh esophageal squamous cell carcinoma (ESCC) (**A**) Significant correlation was observed between the density of M2 macrophages in Kazakh ESCCs tumor islet and stroma (*r* = 0.422; *P* < 0.001). (**B**) and (**C**) Significant correlation was observed between M2 macrophages in Kazakh ESCCs tumor islet, stroma and the expression of MMP9 (*r* = 0.374, *r* = 0.455; *P* < 0.001). (**D** and **E**) Significant correlation between the density of M2 macrophages in Kazakh ESCCs tumor stroma and MVD( *r* = 0.231; *P* < 0.05), but not between the density of M2 macrophages in Kazakh ESCCs tumor islets and MVD( *r* = 0.101; *P* > 0.05), (**F**) significant correlation between the density of the expression of MMP9 and MVD in Kazakh ESCCs .

To explore whether M2 macrophages promote the invasion, metastasis and angiogenesis of Kazakh ESCC by mediating MMP9, we investigated the relationship between the density of M2 macrophages, MVD and the expression of MMP9. Spearman's rank analysis demonstrated positive correlation between MVD and the density of M2 macrophages in tumor stroma(*r* = 0.231; *P* < 0.05). Similar, the expression of MMP9 and the density of M2 macrophages in tumor stroma(*r* = 0.455; *P* < 0.001) and islets(*r* = 0.374; *P* < 0.001) also showed positive correlation respectively (Figure 3C, 3D and 3E). Meanwhile, MVD showed positively association with the expression of MMP9 in Kazakh ESCCs (*r* = 0.292; *P* < 0.05; Figure 3F).

### Association of CD163-positive M2 macrophages with Kazakh ESCC Survival time

**Figure 4:**
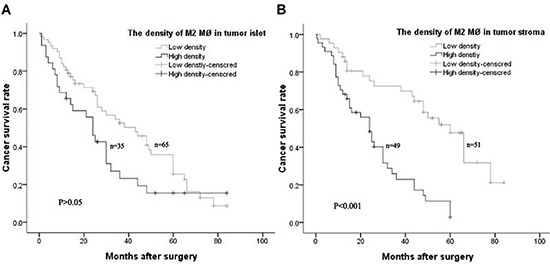
Kaplan–Meier overall survival curves of Kazakh esophageal squamous cell carcinoma (ESCC) patients stratified by the density of CD163 positive M2 macrophages (MØ) in tumor islet and stroma based on the median number (**A**) No significant difference for overall survival between Kazakh patients with high and low density of CD163 positive M2 macrophages (MØ) in tumor islets (*P* > 0.05). (**B**) Patients with high density of CD163 positive M2 macrophages (MØ) in tumor stroma had a poor overall survival (*P* < 0.001).

To investigate the association of M2 macrophages with Kazakh ESCC prognosis, we divided Kazakh ESCC patients into two groups based on the median density of M2 macrophages, high and low density of M2 macrophages. Kaplan–Meier survival curves were used to investigate the influence of M2 macrophages to ESCC patients overall survival (OS) time. We found there was a significant negative correlation between the OS and the density of M2 macrophages in tumor stroma of Kazakh ESCC patients (*P* < 0.001), but not with the density of M2 macrophages in tumor islet (*P* > 0.05, Figure 4).

Univariate Cox proportional hazard analysis revealed lymph node metastasis(*HR* = 15.629), later clinical stage (*HR* = 22.352) and the high density of M2 macrophages in tumor stroma (*HR* = 18.521) were poor prognositic factors for the patients of Kazakh ESCC (all *P* < 0.001, Table 6). Moreover, after Multivariate Cox proportional hazard analysis, we found high-density of M2 macrophages in tumor stroma could serve as an independent prognostic factor for the patients of Kazakh ESCCs (*HR* = 5.464, *P* < 0.05, Table 6).

**Table 6:** Univariate and multivariate analysis of clinicopathological characteristics and M2 macrophages (MØ) with overall survival for Kazakh esophageal carcinoma (ESCC)

Variable	Cases (*N*)	Univariate analysis				Multivariate analysis			
		HR	95% CI		*P*	HR	95% CI		*P*
Age (> 58 y/ ≤ 58 y)	100	0.439	0.979	1.044	0.508				
Sex (female/male)	100	0.067	0.528	1.633	0.796				
Histologic grade(moderate+poor/well)	100	1.050	0.561	1.199	0.305				
Depth of invasion (T3 + T4/T1 + T2)	100	1.528	0.820	2.408	0.216				
Nodal metastasis (positive/negative)	100	15.629	1.757	5.319	0.000*	0.085	0.469	2.777	0.770
TNM stage (III + IV/I + II)	100	22.352	2.156	6.399	0.000*	4.347	1.054	5.508	0.037*
The density of M2 MØ in nest (high/low)	100	4.540	0.976	2.655	0.057	0.064	0.519	1.658	0.801
The density of M2 MØ in tumor stroma (high/low)	100	18.521	1.969	6.114	0.000*	5.464	1.141	4.506	0.019*

^*^P < 0.05.

## DISCUSSION

M2 macrophages are prominent stromal cells that play an important role in tumor growth, angiogenesis and metastasis. Several studies also showed higher density of M2 macrophages in tumor islets and stroma have close relationship with tumor progression and poor prognosis [11, 14]. Nevertheless, the precise role of M2 macrophages in occurrence and progression of Kazakh esophageal carcinoma has yet to be elucidated.

CD163 is confirmed to be a phenotypic marker of M2 macrophages that can be used to distinguish M2 and M1 macrophages. In this study, we found that CD163-positive M2 macrophages were primarily located in the tumor stroma, a few also distrbuted in the tumor islet of Kazkah ESCCs. The density of M2 macrophages in the tumor islet and stroma was significantly higher than that in corresponding CAN tissues, and the increased number of M2 macrophages in stroma was positively associated with more malignant phenotypes including lymph node metastasis and clinical stage progression of Kazakh ESCCs. Such results are similar to those reported in gastric cancers, oral carcinoma and endometrial adenocarcinoma [16–19]. Further study we found high-density of M2 macrophages infiltrated in tumor stoma could be as an independent poorly prognostic factor for Kazakh ESCC evaluation. He and Clear's studies also showed that a high-density of macrophages was associated with worse prognosis in oral cancer and Follicular lymphoma [20, 21]. Prompt increased numbers of M2 macrophages, especially in tumor stroma, were closely related to the occurrence and progression of Kazakh ESCCs, and could be served as an independent poorly prognostic factor for Kazakh ESCCs.

Widespread acceptance, angiogenic vessels developing in the primary tumor are structurally abnormal and functionally immature [22], which provides not only the nutrients and oxygen for tumor growing, but also formed the vascular networks to sustain active intravasation of tumor cells and conduits for tumor cells metastasis. In human cancer tissues, the most common method for semiquantitative evaluation of angiogenesis is to measure microvessel density (MVD) using endothelial markers [23], and which is recognized as one of the most useful prognostic markers for tumor progression and survival in multitudinous cancer patients [24–26]. In this study, CD34 as marker of endothelial cells was used to assess MVD in ESCC tissues. We found the mean value of MVD in tumor was significantly higher compared to corresponding CAN tissues, similar to classify of M2 macrophages density, high MVD in tumor was significantly associated with more malignant phenotypes including lymph node metastasis and clinical stage progression. Then we analyzed the correlation between MVD and the density M2 macrophages, and found the increased MVD was closely related to the increased density of M2 macrophages in tumor stroma. The results are similar to Manabu's in ESCCs study [27]. Bailey also recently identified that increased MVD correlated with higher-density of tumor associated macrophages, play an important role in colorectal cancer progression [28]. These studies may suggest that M2 macrophages are critical for tumor angiogenesis, promoting metastasis and progression of tumor in Kazakh ESCCs.

In general, MMP-9-positive tumor-associated macrophages (TAMs) concomitant with the “restoration” of tumor angiogenesis and metastasis in genetically-deficient tumor bearers transplanted with wild-type hematopoietic cells [29], imply that MMP9 may be critical regulatory factor for macrophages to promote angiogenesis and sustain active of tumor invasion and metastasis. So, we evaluated the expression of MMP9 in ESCCs, found that MMP9 was mainly expressed in stroma cells, only a small section of tissue showed tumor cells staining, and MMP9 expression was much higher in ESCCs than in CANs, particularly in the strong MMP9 staining positive cases. Furthermore, high expression of MMP9 was significantly associated with invasion depth, lymph node metastasis and later clinical stage of Kazakh ESCCs. These results were consistent with previous esophageal carcinoma studies [30–32] . To explore whether M2 macrophages promote the invasion, metastasis and angiogenesis of Kazakh ESCCs by mediating of MMP9, we investigated the relationship between the density of M2 macrophages, MVD and the expression of MMP9, and we found significant correlation between MMP9 expression and the density of M2 macrophages either in the ESCC tumor islets or tumor stroma. We also found increased MVD was correlated with higher expression of MMP9 in Kazakh ESCC tumor tissues. Similarly, El-Kenawy 's study also showed MMP9 positively correlated with MVD in esophageal cancer [30]. These results suggest the M2 macrophages may promote angiogenesis by means of MMP9, and high density M2 macrophages and high expression of MMP9 reflect strong aggressiveness and activity of cancer lesions. Moreover, the results were also similar to some tumor vitro studies, genetic ablation of MMP9 in tumor recipients, resulting in decreased MVD in developing tumors and even preventing the angiogenic switch during cancer progression, provided original evidence for the functional involvement of host MMP9 in tumor angiogenesis [12]. Specific ablation of MMP9 positive TAMs with zoledronic acid resulted in reduced tumor angiogenesis, leading to a conclusion that TAMs deliver angiogenesis-inducing MMP9 are implicated in invasion-promoting processes such as flicking of the angiogenic switch [33, 34]. Combinating the research above with our study, we prompted that M2 macrophages induced MMP9 secretion and promoted tumor aggressiveness and angiogenesis, which is closely associated with the occurrence and progression of Kazakh ESCCs.

## MATERIALS AND METHODS

### Ethics statement

All participants were recruited from the Yili Friendship Hospital in Xinjiang, China. Each participant provided written, informed consent before enrolling in this study. Protocols were approved by the institutional ethics committee of Yili Friendship Hospital in accordance with Helsinki Declaration ethical guidelines.

### Study population

A total of 200 surgically resected and paraffin-embedded human tissues were collected, including 100 Kazakh ESCC tissues and 100 Kazakh cancer adjacent normal tissues (CANs), from the Department of Pathology at Yili Friendship Hospital in Xinjiang, China (collected from 2008 to 2014). The patients consisted of 68 men and 32 women with age range of 33–76 years old and mean age of 58 years old, all had been diagnosed with ESCC, but none had received radiotherapy or chemotherapy before surgery. CAN group participants consisted of 68 men and 32 women with age range of 33–73 years old and mean age of 58 years old. All specimens were sectioned into 5 μm slices and subjected to conventional hematoxylin and eosin staining. A diagnosis of ESCC was confirmed by two pathologists following the World Health Organization histological tumor classification criteria [35]. There were 29 cases of well-differentiated ESCC, 47 cases of moderately differentiated ESCC, and 24 cases of poorly differentiated ESCC. From these, 35 and 65 cases exhibited invasion depths of T1–T2 and T3–T4, respectively. There were 49 cases with lymph node metastasis, 62 cases in clinical stages I–II, and 38 cases in clinical stages III–IV. CAN specimens, which were sampled more than 5 cm away from the cancer region, were confirmed to be free of cancer tissue. The survival status of all patients was followed up by telephone contact until December 2015. The median follow-up for living patients was 30 months (range, 1–84 months). Overall survival (OS) was defined as the interval between surgery and death or between surgery and the last follow-up for surviving patients. Among the 100 patients who were recruited, 43 (43.0%) died, and 57 (57.0%) remained alive during the follow-up period.

### Immunohistochemistry

Paraffin-embedded tissue samples were cut into 4 μm-thick sections and mounted on polylysine-coated slides. Samples were dewaxed in xylene and rehydrated using a graded series of ethanol solutions. After deparaffinization, endogenous peroxidase activity was blocked by incubation in a 3% peroxide-methanol solution at room temperature (RT) for 10 mins, and then antigen retrieval was performed at 100°C in an autoclave for 7 mins. Samples were then incubated at RT for 30 mins. Afterwards, sections were washed with phosphate-buffered saline (PBS) three times for 5 mins each time. They were then incubated with mouse anti-human CD163 antigen monoclonal antibody (clone 10D6, Zhongshan Goldenbridge Biotechnology Co., LTD., Beijing, China), mouse anti-human MMP9 antigen monoclonal antibody (clone Sc-2c3, Santa Cruz Biotechnology, Santa Cruz, CA, USA) and mouse anti-human CD34 antigen monoclonal antibody (clone QBEnd/10, Zhongshan Goldenbridge Biotechnology Co., LTD., Beijing, China). Thoroughly washing with PBS was then performed, and primary antibody binding was visualized using a DAKO EnVision kit (DAKO, Glostrup, Denmark) following the manufacturer's instructions. Finally, sections were faintly counterstained with hematoxylin and mounted with glycerol gelatin.

### Immunoreactivity evaluation

The numbers of CD163-positive macrophages were analyzed as described previously [36]. Slides were examined by light microscopy and the five most representative hot spots were selected from low-power fields (LPFs, 100×) per slide using an Olympus BX51TF microscope (Olympus, Japan). Tumor islet and stroma areas were defined and the numbers of CD163-positive macrophages were counted in high-power fields (HPFs, 400×) by two pathologists. When cell counts differed by more than 10 cells per HPF, samples would be counted again a week later until recording differences were below 10 counts. The mean number of macrophages per HPF across five hot spots for every sample (tumor islet and tumor stroma) was defined as the M2 macrophages density.

Microvessel was measured by the average number of CD34 positive vessels. Similar to macrophages counts,we choose five most representative hot spots to observe and use a 400× magnification field to count the number of microvessels. Determined microvessel density (MVD) was expressed as the number of stained microvessels per optical field. The vessel was composed by any CD34 positive cell or cell cluster, and the diameter less than eight red blood cells was counted as a microvessel, as described in the Weidner method [37]. When microvessel counts differed by more than 10 per HPF, samples would be counted again a week later until recording differences were below 10 counts. The mean number of microvessel per HPF across five hot spots for every sample (tumor islet and tumor stroma) was defined as the MVD.

MMP9 immunohistochemistry (IHC) reactivity was evaluated following previously described methods [38, 39]. Positive IHC stains were defined as yellow-brown color following the manufacturer's guidelines (Figure 3). IHC staining slides were scored as positive or negative by percentage and intensity of positive cells, where the scoring percentage of positively stained cells was as follows: 0 = < 5%, 1 = 6% – 25%, 2 = 26% – 50%, 3 = 51% – 75%, and 4 = 76% – 100%; staining intensity scoring was: 0 = absent, 1 = weak, 2 = moderate, and 3 = strong. A final score was based on multiplying both scores from individual slides (Supplementary Table 1), where: 0–1 was negative (−), 2–3 was weak positive (1+), 4–6 was moderate positive (2+), and 8–12 was strong positive (3+).

### Statistical analysis

SPSS version 13.0 software (IBM, Endicott, NY) was used for all statistical analyses. The Mann-Whitney nonparametric test was used to compare the density of M2 macrophages and MVD between ESCC and CAN groups. For categorical analysis, the median value of M2 macrophages density and MVD were used as a cut-off point to dichotomize the continuous variables. The X^2^ test was adopted for analysis of correlations between the expression of MMP9 and clinicopathological parameters of ESCCs. Spearman's rank correlation method was used to evaluate the correlations between the density of M2 macrophages, MVD and MMP9 expression. Survival curves were estimated by the Kaplan Meier method. The correlation between the density of M2 macrophages, clinicopathological parameters and overall survival (OS) rates of Kazakh ESCCs was evaluated in univariate analysis, and was further analyzed with multivariate analysis by using Cox proportional hazard regression model. *P*-values were calculated by using the Epi-Info program, and *P*-values < 0.05 were considered significant.

## SUPPLEMENTARY MATERIALS FIGURES AND TABLES



## Abbreviations

MØ: macrophages
ESCC: esophageal squamous cell carcinoma
CAN: cancer adjacent normal
MMPs: matrix metalloproteinases
MVD: microvessel density
interferon-γ: IFN-γ
tumor necrosis factor alpha: TNF-α
histocompatibility complex: MHC
interleukin-6: IL-6
TAM: tumor associated macrophage
IHC: immunohistochemistry

## References

[1] Parkin DM, Bray F, Ferlay J, Pisani P (2005). Global cancer statistics, 2002. CA Cancer J Clin.

[2] Ya MZ (1988). The distribution of esophageal cancer in Xinjiang. J Xinjiang Med Univ (China).

[3] Ekman S, Dreilich M, Lennartsson J, Wallner B, Brattstrom D, Sundbom M, Bergqvist M (2008). Esophageal cancer: current and emerging therapy modalities. Expert Rev Anticancer Ther.

[4] Lee CH, Liu SY, Chou KC, Yeh CT, Shiah SG, Huang RY, Cheng JC, Yen CY, Shieh YS (2014). Tumor-associated macrophages promote oral cancer progression through activation of the Axl signaling pathway. Ann Surg Oncol.

[5] Zhang BC, Gao J, Wang J, Rao ZG, Wang BC, Gao JF (2011). Tumor-associated macrophages infiltration is associated with peritumoral lymphangiogenesis and poor prognosis in lung adenocarcinoma. Med Oncol.

[6] Gwak JM, Jang MH, Kim DI, Seo AN, Park SY (2015). Prognostic value of tumor-associated macrophages according to histologic locations and hormone receptor status in breast cancer. PLoS One.

[7] Gordon S, Taylor PR (2005). Monocyte and macrophage heterogeneity. Nat Rev Immunol.

[8] Mantovani A, Sozzani S, Locati M, Allavena P, Sica A (2002). Macrophage polarization: tumor-associated macrophages as a paradigm for polarized M2 mononuclear phagocytes. Trends Immunol.

[9] Mantovani A, Sica A (2010). Macrophages, innate immunity and cancer: balance, tolerance and diversity. Curr Opin Immunol.

[10] Sica A, Allavena P, Mantovani A (2008). Cancer related inflammation: the macrophage connection. Cancer Lett.

[11] Qian BZ, Pollard JW (2010). Macrophage diversity enhances tumor progression and metastasis. Cell.

[12] Bergers G, Brekken R, McMahon G, Vu TH, Itoh T, Tamaki K, Tanzawa K, Thorpe P, Itohara S, Werb Z, Hanahan D (2000). Matrix metalloproteinase-9 triggers the angiogenic switch during carcinogenesis. Nat Cell Biol.

[13] Obeid E, Nanda R, Fu YX, Olopade OI (2013). The role of tumor-associated macrophages in breast cancer progression (review). Int J Oncol.

[14] Pollard JW (2004). Tumour-educated macrophages promote tumour progression and metastasis. Nat Rev Cancer.

[15] Deryugina EI, Quigley JP (2015). Tumor angiogenesis: MMP-mediated induction of intravasation- and metastasis-sustaining neovasculature. Matrix Biol.

[16] Go Y, Tanaka H, Tokumoto M, Sakurai K, Toyokawa T, Kubo N, Muguruma K, Maeda K, Ohira M, Hirakawa K (2016). Tumor-Associated Macrophages Extend Along Lymphatic Flow in the Pre-metastatic Lymph Nodes of Human Gastric Cancer. Ann Surg Oncol.

[17] Park JY, Sung JY, Lee J, Park YK, Kim YW, Kim GY, Won KY, Lim SJ (2016). Polarized CD163+ tumor-associated macrophages are associated with increased angiogenesis and CXCL12 expression in gastric cancer. Clin Res Hepatol Gastroenterol.

[18] Hu Y, He MY, Zhu LF, Yang CC, Zhou ML, Wang Q, Zhang W, Zheng YY, Wang DM, Xu ZQ, Wu YN, Liu LK (2016). Tumor-associated macrophages correlate with the clinicopathological features and poor outcomes via inducing epithelial to mesenchymal transition in oral squamous cell carcinoma. J Exp Clin Cancer Res.

[19] Kubler K, Ayub TH, Weber SK, Zivanovic O, Abramian A, Keyver-Paik MD, Mallmann MR, Kaiser C, Serce NB, Kuhn W, Rudlowski C (2014). Prognostic significance of tumor-associated macrophages in endometrial adenocarcinoma. Gynecol Oncol.

[20] He KF, Zhang L, Huang CF, Ma SR, Wang YF, Wang WM, Zhao ZL, Liu B, Zhao YF, Zhang WF, Sun ZJ (2014). CD163+ tumor-associated macrophages correlated with poor prognosis and cancer stem cells in oral squamous cell carcinoma. Biomed Res Int.

[21] Clear AJ, Lee AM, Calaminici M, Ramsay AG, Morris KJ, Hallam S, Kelly G, Macdougall F, Lister TA, Gribben JG (2010). Increased angiogenic sprouting in poor prognosis FL is associated with elevated numbers of CD163+ macrophages within the immediate sprouting microenvironment. Blood.

[22] Carmeliet P, Jain RK (2011). Principles and mechanisms of vessel normalization for cancer and other angiogenic diseases. Nat Rev Drug Discov.

[23] Wu L, Li X, Ye L, Shayiremu D, Deng X, Zhang X, Jiang W, Yang Y, Gong K, Zhang N (2014). Vascular endothelial growth inhibitor 174 is anegative regulator of aggressiveness and microvascular density in human clear cell renal cell carcinoma. Anticancer Res.

[24] Ammendola M, Sacco R, Marech I, Sammarco G, Zuccala V, Luposella M, Patruno R, Giordano M, Ruggieri E, Zizzo N, Gadaleta CD, Ranieri G (2015). Microvascular density and endothelial area correlate with Ki-67 proliferative index in surgically-treated pancreatic ductal adenocarcinoma patients. Oncol Lett.

[25] Guo Y, Xia P, Zheng JJ, Sun XB, Pan XD, Zhang X, Wu CZ (2015). Receptors for advanced glycation end products (RAGE) is associated with microvessel density and is a prognostic biomarker for clear cell renal cell carcinoma. Biomed Pharmacother.

[26] Miyata Y, Ohba K, Matsuo T, Watanabe S, Hayashi T, Sakai H, Kanetake H (2013). Tumor-associated stromal cells expressing E-prostanoid 2 or 3 receptors in prostate cancer: correlation with tumor aggressiveness and outcome by angiogenesis and lymphangiogenesis. Urology.

[27] Shigeoka M, Urakawa N, Nakamura T, Nishio M, Watajima T, Kuroda D, Komori T, Kakeji Y, Semba S, Yokozaki H (2013). Tumor associated macrophage expressing CD204 is associated with tumor aggressiveness of esophageal squamous cell carcinoma. Cancer Sci.

[28] Bailey C, Negus R, Morris A, Ziprin P, Goldin R, Allavena P, Peck D, Darzi A (2007). Chemokine expression is associated with the accumulation of tumour associated macrophages (TAMs) and progression in human colorectal cancer. Clin Exp Metastasis.

[29] Huang S, Van Arsdall M, Tedjarati S, McCarty M, Wu W, Langley R, Fidler IJ (2002). Contributions of stromal metalloproteinase-9 to angiogenesis and growth of human ovarian carcinoma in mice. J. Natl. Cancer Inst.

[30] M El-Kenawy Ael, Lotfy M, El-Kott A, El-Shahat M (2005). Significance of matrix metalloproteinase 9 and CD34 expressions in esophageal carcinoma: correlation with DNA content. J Clin Gastroenterol.

[31] Gu ZD, Li JY, Li M, Gu J, Shi XT, Ke Y, Chen KN (2005). Matrix metalloproteinases expression correlates with survival in patients with esophageal squamous cell carcinoma. Am J Gastroenterol.

[32] Li Y, Guo H, Dong D, Wu H, Li E (2013). Expression and prognostic relevance of cyclophilin A and matrix metalloproteinase 9 in esophageal squamous cell carcinoma. Diagn Pathol.

[33] Giraudo E, Inoue M, Hanahan D (2004). An amino-bisphosphonate targets MMP-9-expressing macrophages and angiogenesis to impair cervical carcinogenesis. J Clin Invest.

[34] Rogers TL, Holen I (2011). Tumour macrophages as potential targets of bisphosphonates. J Transl Med.

[35] Li ZS, Li Q (2011). [The latest 2010 WHO classification of tumors of digestive system] [Article in Chinese]. Zhonghua Bing Li Xue Za Zhi.

[36] Shabo I, Olsson H, Sun XF, Svanvik J (2009). Expression of the macrophage antigen CD163 in rectal cancer cells is associated with early local recurrence and reduced survival time. Int J Cancer.

[37] Weidner N, Semple JP, Welch WR, Folkman J (1991). Tumor angiogenesis and metastasis—correlation in invasive breast carcinoma. N Engl J Med.

[38] Hu JM, Chang AM, Chen YZ, Yuan XL, Li F (2016). Regulatory Role of miR-203 in Occurrence and Progression of Kazakh Esophageal squamous cell carcinoma. Sci Rep.

[39] Hu JM, Li L, Chen YZ, Liu C, Cui X, Yin L, Yang L, Zou H, Pang L, Zhao J, Qi Y, Cao Y, Jiang J (2014). HLA-DRB1 and HLA-DQB1 methylation changes promote the occurrence and progression of Kazakh ESCC. Epigenetics.

