# Supplementary material for: CD163 as a marker of M2 macrophage, contribute to predict aggressiveness and prognosis of Kazakh esophageal squamous cell carcinoma
Source: Oncotarget. 2017 Feb 22;8(13):21526–38. doi: 10.18632/oncotarget.15630 (PMC5400603; doi:10.18632/oncotarget.15630)
Supplement: Supplementary file 1 [file oncotarget-08-21526-s001.pdf]

CD163 as a marker of M2 macrophage, contribute to predicte aggressiveness and prognosis of Kazakh esophageal squamous cell carcinoma

Supplementary Materials

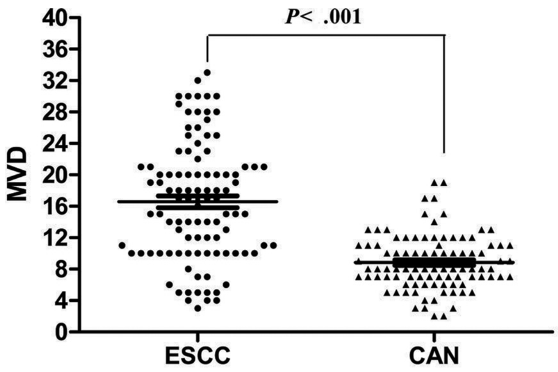

Supplementary Figure 1: The value of microvessel density (MVD) in Kazakh esophageal squamous cell carcinoma (ESCC) was significantly higher compared to Cancer adjacent normal (CAN) tissues.

Supplementary Table 1: Scoring criteria of immunohistochemistry (IHC) assay with MMP-9 antibody used in this study

Staining positive cell		Staining intensity		Final Score product	
**Percent (%)**	**Score 1**	**intensity**	**Score 2**	**Score 1×Score 2**	**Score 3**
< 5%	0	Absent	0	0–1	0 (−)
6%–25%	1	weak	1	2–3	1 (1+)
26%–50%	2	moderate	2	4–6	2 (2+)
51%–75%	3	strong	3	8–12	3 (3+)
75%–100%	4				

Note: IHC staining slides were scored as positive or negative by the percentage and intensity of positive cells Scoring the percent of positively stained cells-Score 1(0 = < 5%; 1 = 6% – 25%; 2 = 26% – 50%; 3 = 51% – 75%; and 4 = 76% – 100%). Scoring the intensity of the staining-Score 2(0 = absent; 1 = weak; 2 = moderate; 3 = strong). The final score was based on multiplying both scores from individual slides (Score 1 × Score 2): 0–1 was negative (−), 2–3 was weak positive (1+), 4–6 was moderately positive (2+), and 8–12 was strong positive (3+).
